# Coumarin-Mediated Growth Regulations, Antioxidant Enzyme Activities, and Photosynthetic Efficiency of *Sorghum bicolor* Under Saline Conditions

**DOI:** 10.3389/fpls.2022.799404

**Published:** 2022-04-06

**Authors:** Robina Sultana, Xiukang Wang, Muhammad Azeem, Tabassum Hussain, Athar Mahmood, Sajid Fiaz, Muhammad Qasim

**Affiliations:** ^1^Biosaline Research Laboratories, Department of Botany, University of Karachi, Karachi, Pakistan; ^2^College of Life Sciences, Yan’an University, Yan’an, China; ^3^Dr. Muhammad Ajmal Khan Institute of Sustainable Halophyte Utilization, University of Karachi, Karachi, Pakistan; ^4^Department of Agronomy, University of Agriculture, Faisalabad, Pakistan; ^5^Department of Plant Breeding and Genetics, The University of Haripur, Haripur, Pakistan

**Keywords:** seed priming, foliar application, growth regulation, oxidative stress, chlorophyll fluorescence

## Abstract

Secondary metabolites, such as phenolic compounds, play an important role in alleviating salinity-induced negative effects in plants. The present study focused on seed priming and foliar application of a potent phenolic compound, coumarin, to induce salinity tolerance in *Sorghum bicolor* var. SS-77. Based on pilot experiment, 100 mg L^−1^ concentration of coumarin was applied to mitigate the negative effects of salinity on Sorghum, grown at 0, 100, and 200 mM NaCl under netted greenhouse conditions. Coumarin was applied to each salinity treatment in four different ways (i) non-primed control (NP), (ii) seed priming (COP), (iii) foliar application (COF), and (iv) a combination of seed priming and foliar application (COPF). Salinity stress significantly reduced the plant growth, biochemical attributes, and photosynthetic efficiency of Sorghum, whereas coumarin treatments (COP, COF, and COPF) showed a significant increase (P< 0.01) in above-mentioned parameters at all salinities. Among all, the combined treatment (COPF) showed maximum increase in growth, biochemicals, photosynthetic pigments, antioxidant enzymes, and photosynthetic efficiency parameters. Therefore, it is suggested that a combination of seed priming and foliar spray of 10 mg L^−1^ coumarin is more suitable than their individual applications. It is an environment friendly and economically feasible approach that will be used to improve salinity tolerance of Sorghum and helpful to get considerable biomass from saline degraded lands to fulfill food, fodder, and energy demands of the ever-growing population.

## Introduction

*Sorghum bicolor* is a moderately salt tolerant, multipurpose C_4_ plant, widely cultivated in arid and semi-arid areas for food, fodder, and biofuel purposes (Hassan et al., 2019). It is a fifth most important food crop, which covers approximately 46 million hectares of land in more than 100 countries with an average annual production of 60 million tons (Iqbal et al., 2010; Appiah-Nkansah et al., 2019). Sorghum has a potential to grow under stress conditions like salinity and drought; however, it requires detailed information at morphological, physiological, and biochemical level (Sun et al., 2012). Salinity stress is one of the major abiotic constraints that threaten plant growth, development, and productivity. Salinization is escalating with an alarming pace, which already affected about 20% of agriculture land around the globe resulting in almost 12 billion USD losses annually (Zulfiqar et al., 2021). It is estimated that approximately 50% arable land will be lost by salinization till 2050 (Ahanger et al., 2017; Machado and Serralheiro, 2017). Most crops are salt sensitive (Ruiz-Lozano et al., 2012). Salinity stress impairs crop growth mainly due to hyper-accumulation of toxic ions (Na^+^ and Cl^−^) resulting specific ion toxicity, osmotic stress, and oxidative damages, which in turn leading to nutritional, hormonal, and enzymatic imbalances. Additionally, salinity stress reduced photosynthetic performance by altering enzyme activities, destroying photosynthetic pigments, reducing leaf area, and inhibiting photosystem efficiency (Hussain et al., 2020a). Higher Na^+^ accumulation antagonizes the K^+^ uptake and causes ionic and metabolic imbalances and triggers oxidative stress by overproduction of reactive oxygen species (ROS) responsible to damage various cellular components such as plasma membranes, proteins, lipids, and nucleic acids (Alamri et al., 2020; Zhang et al., 2020).

In contrast, salinity-tolerant plants display various responses including osmoregulation, ion homeostasis, mineral balance, protection of photosynthetic apparatus, production of secondary metabolites, and activation of antioxidant defense system (El-Esawi et al., 2020; Afsar et al., 2021).

Various approaches have been adopted to mitigate the salinity-induced damages in crop plants, in which exogenous applications through seed priming and foliar spray of phytohormones, osmo-protectants, osmolytes, and antioxidants are considered cost-effective and eco-friendly (Al-Huqail et al., 2020; Azeem et al., 2020). Exogenous application of these compounds such as salicylic acid (Azeem et al., 2019; Ahmad et al., 2020), ascorbic acid, putrescine (Seleem et al., 2021), polyamines (Sagor et al., 2021), proline (Ghafoor et al., 2019), glycine betaine (Hamani et al., 2021), melatonin (Ali et al., 2021), strigolactone (Zulfiqar et al., 2021), and coumarin (Sultana et al., 2020; Parvin et al., 2021) have found effective in improving plants salinity tolerance and biomass production.

Phenolic compounds play important roles during stressful conditions. Phenolic acids, flavonoids, tannins, phenylpropanoids, coumarin, benzoic acid derivatives, lignin, and lignin precursors help in stress resistance by involving in growth and developmental processes of plants (Lattanzio, 2013). Various phenolic compounds including coumarin, ferulic acid, and allagic acid have been reported to increase stress tolerance (Singh et al., 2017). Coumarin (2H-chromen-2-one; COU) is a hydroxycinnamic acid and amongst the common phenolic compounds in plants. It works as a free radical scavenger, membrane stabilizer, and inhibitor of lipid peroxidation that promote plant growth and development (Siwinska et al., 2018). Strong antioxidant, antimicrobial, and cytotoxic properties of this compound have been reported (Saleh et al., 2015). Studies indicated that exogenous application of COU enhanced the antioxidant performance, levels of phenolic compounds, activities of major metabolic enzymes, and ions homeostasis of different crop plants under stressful conditions (Parvin et al., 2020). In our pervious study, we reported that priming of Sorghum seeds with 50 and 100 mg L^−1^ COU effectively mitigate the negative effects of salinity by improving antioxidant enzyme activities at seed germination and early seedling establishment phases (Sultana et al., 2020). Based on the previous findings, present study focused on different modes of coumarin application including (1) seed priming, (2) foliar spray, and (3) a combination of seed priming and foliar spray to analyze the most effective mode of treatment to alleviate the negative effects of salinity on Sorghum. The COU-mediated stimulation in physio-biochemical, antioxidant, and photosynthetic responses as well as their relationship with overall plant growth performance were also assessed.

## Materials and Methods

### Experimental Setup

In our previous experiment, we used 50 and 100 mg L^−1^ COU for seed priming of *Sorghum bicolor*; however, the 100 mg L^−1^ COU showed promising results (Sultana et al., 2020), hence selected for this study. Ten seeds of Sorghum were sown in each plastic pot (14.5 cm diameter and 12 cm height) containing 2 kg of acid-washed quartz sand and half-strength Hoagland’s nutrient solution was provided through sub-irrigation. After the two-leaf stage (15th day of emergence), seedlings were thinned to three seedlings per pot of equal size and vigor. After thinning, plants were treated with 0, 100, and 200 mM NaCl solutions in a netted greenhouse under ambient conditions high temperature (34–38°C), low temperature (24–28°C), RH at 12 noon (55–60%), photoperiod (13–13.5 h), and PPFD at 12 noon (1,400–1,600 μmol m^−2^ s^−1^). To avoid the osmotic shock, NaCl concentration was increased gradually (50 mM NaCl per day) until the final concentration reached. The COU application in each salinity treatment was applied in four different ways, i.e., (1) non-primed control (NP), (2) seed priming (COP), (3) foliar spray (COF), and (4) a combination of seed priming + foliar spray (COPF) with six replicates each. The COP and COPF seeds were primed with 100 mg L^−1^ coumarin, whereas the NP and COF treatments remained unprimed. COF and COPF were sprayed with 100 mg L^−1^ coumarin three times during the experiment. First foliar spray was done at 20th day of seedling emergence and rests were done after 1-week interval of first spray. After 40 days of growth, plants were harvested and growth and biochemical parameters were measured.

### Estimation of Photosynthetic Pigments

Fresh sample (0.25 g) of Sorghum leaves was ground with liquid nitrogen, homogenized in 5 ml of 80% acetone and centrifuged at least three times at 3,000 rpm for 10 min each. Supernatant then separated and absorbance was measured at 645, 663, and 480 nm to calculate the chlorophyll *a*, chlorophyll *b*, total chlorophylls, and carotenoids (Kirk and Allen, 1965).

Total anthocyanin content was estimated using fresh leaves (0.25 g) from each treatment, homogenized with 5 ml of 0.1 N methanol-HCl reagents, and centrifuged at 4,000 rpm for 10 min (Ganjewala et al., 2010). Supernatant was then collected and absorbance was recorded at 537 and 657 nm.

### Estimation of Chlorophyll Fluorescence

A pulse-modulated chlorophyll fluorescence meter (PAM 2500, Walz, Germany) was used to evaluate the photosynthetic performance of plants. Healthy fully expanded leaves from the third and fourth nodes were selected and adapted in dark for 30 min before measuring the lower range of the fluorescence (Fo; Baker and Rosenqvist, 2004) in which light of a specific wavelength and specific intensity of photons (<0.1 μmol photon m^−2^ s^−1^) was measured. Afterward, leaves were subjected to 10,000 photons (μmol m^−2^ s^−1^) light for 0.8 s to analyze the maximum fluorescence (Fm; Kitajima and Butler, 1975). Furthermore, the leaves were continuously subjected to the light to calculate the steady state (Fs) and maximum florescence (Fm). The effective photochemical quantum yield of PSII as Fm′-Fs/Fm′ (Genty et al., 1989), quantum yield of regulated non-photochemical energy loss in PSII [Y (NPQ) = (Fs/Fm′) – (Fs/Fm)], and quantum yield of non-regulated non-photochemical energy loss in PSII [Y(NO) = Fs/Fm] were calculated (Kramer et al., 2004). Non-photochemical quenching of fluorescence (NPQ), which is proportional to the rate of constant heat dissipation (Bilger and Björkman, 1990), was calculated (as NPQ = Fm/Fm′^−1^). Coefficient of photochemical quenching (qP) was calculated as (Fm′-Fs)/(Fm′-Fo′; Schreiber et al., 1986). PSII is used to calculate the linear electron transport rate (ETR; Krall and Edwards, 1992), ETR = PSII * PPFD * 0.5 * 0.84, where the photosynthetic photon flux density (PPFD) is incident on the leaf, 0.5 is a factor that adopts an equal distribution of energy between the two photosystems and 0.84 is assumed to be the leaf absorbance.

### Estimation of Total Phenolic Content and Total Soluble Carbohydrates

Total phenolic content (TPC) was measured in dry plant material (Singleton and Rossi, 1965). Plant dry material (0.1 g) was homogenized in 10 ml of 80% methanol and centrifuged at 3,000 rpm for 10 min. Extracted supernatant was diluted and 100 μl of the extract was mixed with 500 μl of 0.2 N Folin–Ciocalteu reagents and incubated for 5 min. Saturated sodium carbonate (75 g L^−1^, 400 μl) solution was added, and the mixture was further incubated for 1.5 h at room temperature. Absorbance was recorded at 765 nm against the standard curve of gallic acid. Total soluble carbohydrates were estimated according to the method of Yemm and Willis (1954). Water extract (10 ml) of dried leaves (0.1 g) was boiled for 1 h. The extract was cooled and filtered and 1 ml hot water extract mixed with 5 ml of anthrone reagent, boiled in a water bath for 30 min. After cooling, the absorbance was measured at 620 nm against the standard curve of glucose.

### Estimation of H_2_O_2_ and Malondialdehyde Contents

Fresh sample (0.5 g) of Sorghum leaves was ground in liquid nitrogen, homogenized in 3% ice-cold trichloro-acetic acid (TCA), and centrifuged at 15,000 rpm for 15 min at 4°C (Velikova et al., 2000). Supernatant (hereafter TCA extract) was stored at −20°C. For estimation of H_2_O_2_, 0.5 ml TCA extract was mixed with 0.5 ml potassium phosphate buffer (pH 7.0, 0.5 ml) and 1 ml potassium iodide (1 M) and incubated in dark. After 10 min, the absorbance was recorded at 390 nm.

For estimation of MDA, 0.5 ml TCA extract was mixed with 0.5 ml 20% (w/v) trichloro-acetic acid containing 2-thiobarbituric acid (0.5%) in a capped glass test tube and placed in a water bath at 95°C for 30 min (Heath and Packer, 1968). The reaction was terminated by placing the sample in ice bath and further centrifuged at 12,000 × *g* for 10 min at 4°C. Absorbance was recorded at 532 nm, 600 nm, and 450 nm.

### Estimation of Proteins and Antioxidant Enzyme Activities

Fresh samples (0.5 g) of Sorghum leaves were ground in mortar and pestle using liquid nitrogen and homogenized in 5 ml of potassium phosphate buffer (100 mM, pH 7.5). The solutions were centrifuged at 20,000 rpm at 4°C for 20 min. The supernatants were carefully transferred into Eppendorf tubes and stored at −40°C.

Total soluble proteins were estimated according to the method described by Bradford (1976), against the standard curve of bovine serum albumin.

Catalase (CAT) activity (extinction coefficient = 39.4 M^−1^ cm^−1^) was measured according to Aebi (1984). Reaction mixture (3 ml) containing potassium phosphate buffer (50 mM, pH = 7.0), 25 mM H_2_O_2_ and 100 μl of enzyme extract was placed in a quartz cuvette in a spectrophotometer, and decrease in absorbance was recorded at 240 nm for 1 min.

Ascorbate peroxidase (APX) activity (extinction coefficient = 2.8 mM^−1^ Cm^−1^) was estimated according to the method of Nakano and Asada (1981). Reaction mixture (3 ml) containing potassium phosphate buffer (100 mM, pH 7.0), 0.5 mM ascorbic acid, 0.1 mM H_2_O_2_, and 100 μl of enzyme extract was placed in a quartz cuvette in a spectrophotometer and linear decrease in absorbance was recorded at 290 nm for 1 min.

Guaiacol peroxidase (GPX) activity (extinction coefficient = 26.6 mM^−1^ Cm^−1^) was estimated according to the method of Polle et al. (1994). Reaction mixture (3 ml) containing potassium phosphate buffer (100 mM, pH 7.0), 20 mM guaiacol, 10 mM H_2_O_2_, and 50 μl of enzyme extract was placed in a quartz cuvette in a spectrophotometer and increase in absorbance was recorded at 470 nm for 1 min. The specific enzyme activities were determined by dividing the enzyme activity (unit mL^−1^ enzyme) of protein concentration (mg mL^−1^) of the respective plant samples, and presented as the unit mg^−1^ protein.

### Statistical Analysis

The data are presented as the mean ± S.E. Statistical analyses were carried out using SPSS Ver. 20.0 for Windows (SPSS Inc., Chicago, IL, United States; IBM SPSS 2012). Two-way ANOVA and Tukey’s HSD test were performed to analyze the differences (*p* < 0.05) among treatments, and the ANOVA was performed by the least significant difference (LSD) test to compare the significance of priming and salinity treatments.

## Results

### Growth Responses

**Figure 1:**
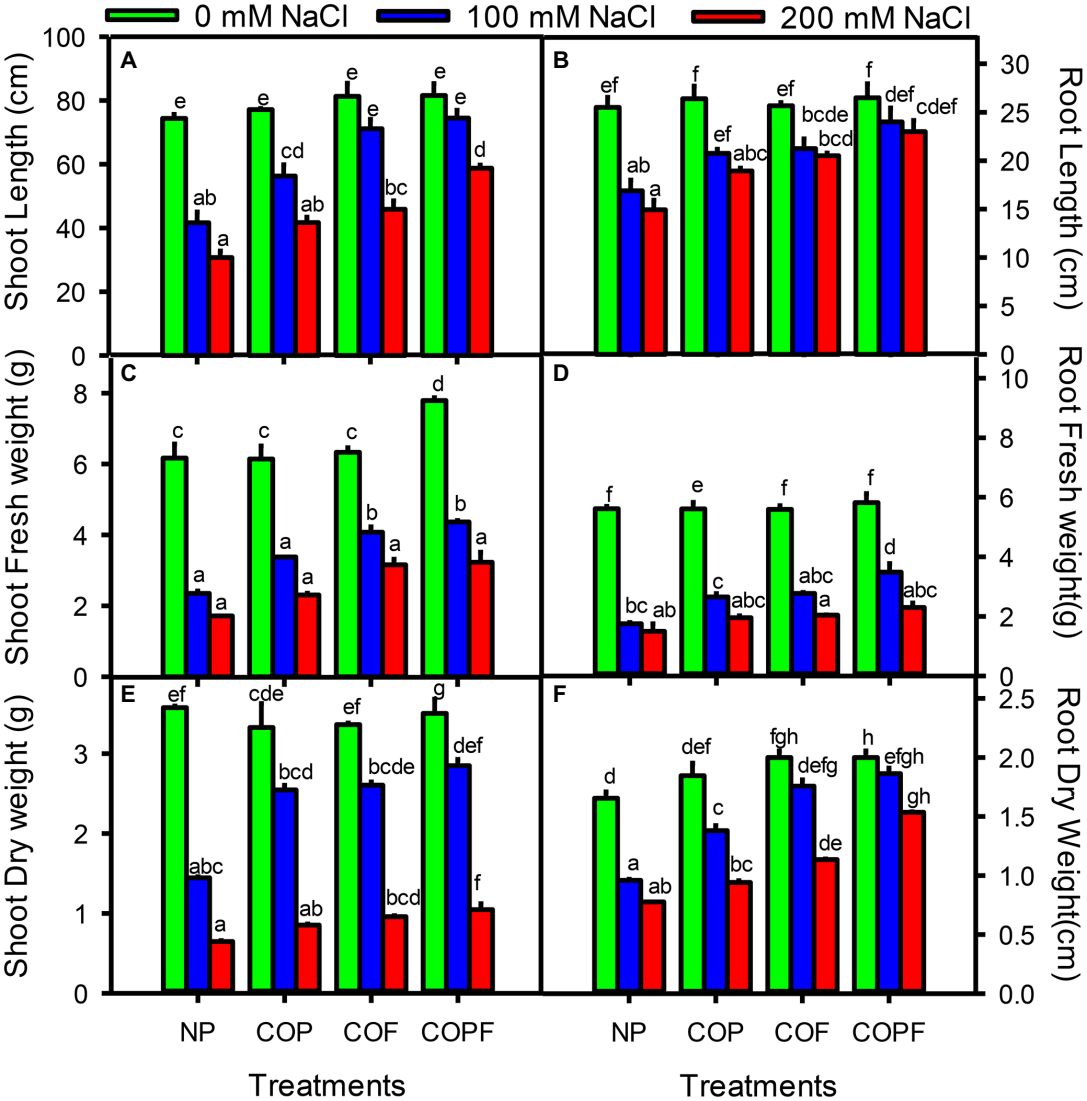
Effect of salinity stress and coumarin applications on growth parameters [Shoot length **(A)**, Root length **(B)**, Shoot fresh weight **(C)**, Root fresh weight **(D)** Shoot dry weight **(E)** and Root dry weight **(F)**]. Plants were treated with 0 mg L^−1^ coumarin (NP), 100 mg L^−1^ coumarin as seed priming (COP), 100 mg L^−1^ coumarin as foliar application (COF) and combined treatment (COPF), under 0, 100 and 200 mM NaCl. Values are represented as means ±SE (*n* = 3). Different letters represent significant differences (*p* < 0.05) by Tukey’s HSD test.

Plant growth parameters in terms of lengths and fresh and dry weights of shoot and root of Sorghum were decreased significantly (*p* < 0.01) with increasing salinity. However, all COU applications (COP, COF, and COPF) significantly increased (*p* < 0.01) the lengths and fresh and dry weights of shoot and root at all salinities as compared to the non-primed control. The highest increase in length and biomass were observed in the COPF treatment under both non-saline and saline conditions (Figures 1A–F).

### Photosynthetic Pigments and Chlorophyll Fluorescence

**Figure 2:**
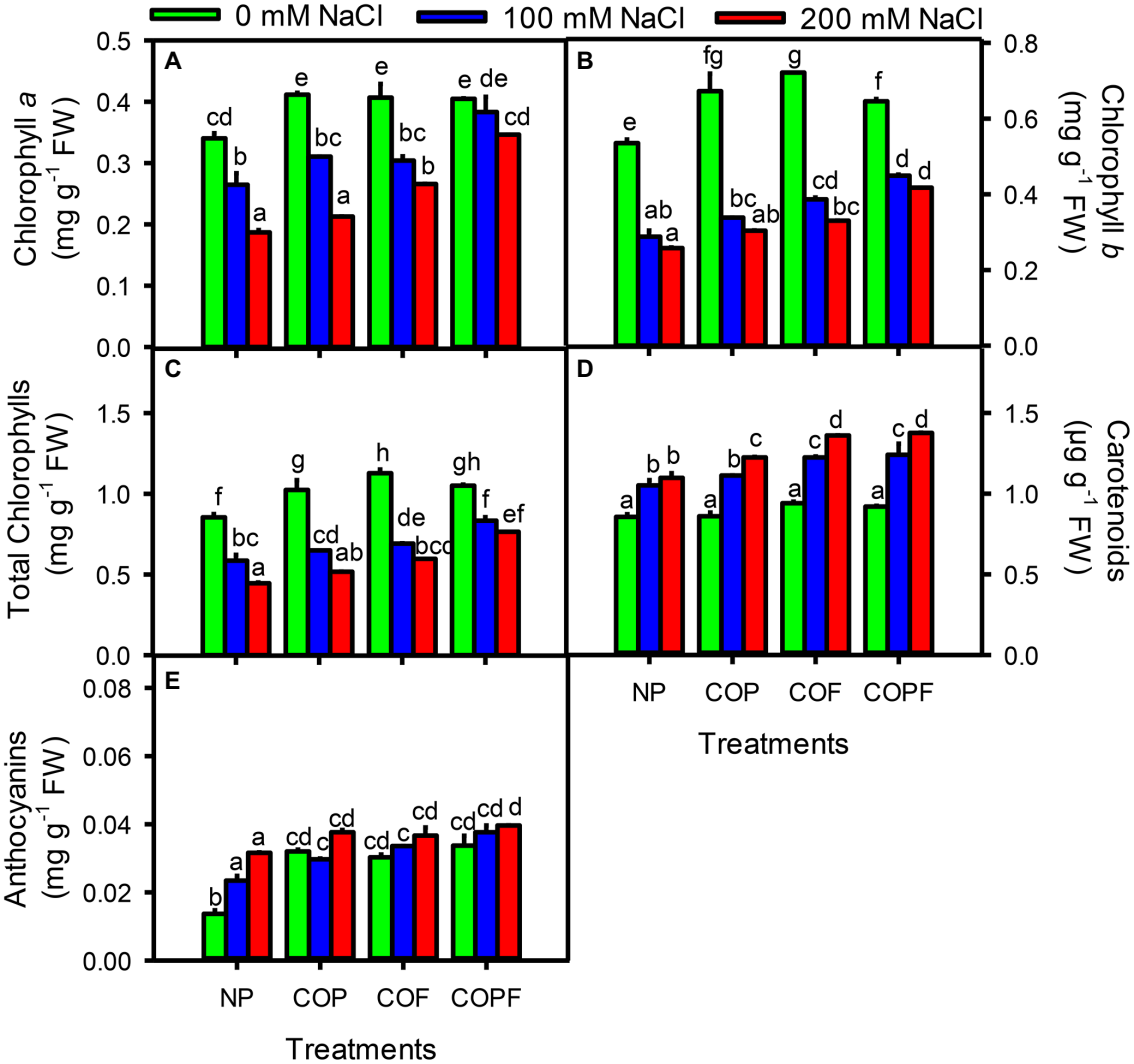
Effect of salinity and coumarin applications on Photosynthetic Pigments. Chlorophyll *a* **(A)**, Chlorophyll *b* **(B)**, Total chlorophylls **(C)**, Carotenoids **(D)** and Anthocyanins **(E)**. Plants were treated with 0 mg L^−1^ coumarin (NP), 100 mg L^−1^ coumarin as seed priming (COP), 100 mg L^−1^ coumarin as foliar application (COF) and combined treatment (COPF), under 0, 100 and 200 mM NaCl. Values are represented as means ±SE (*n* = 3). Different letters represent significant differences (*p* < 0.05) by Tukey’s HSD test.

The chlorophylls (*chl a*, *chl b*, and total chlorophylls) significantly decreased (*p* < 0.01) with increasing salinity. However, COU application showed an increasing trend under saline conditions compared to their respective counterparts. Furthermore, carotenoids and anthocyanins were directly proportional to increasing salinity. However, anthocyanins and carotenoids increased to a greater extent in all coumarin treatments than the control. Anthocyanins and carotenoids were higher in the COPF treatment than the non-primed and other COU treatments at 200 mM NaCl (Figures 2A–E).

**Figure 3:**
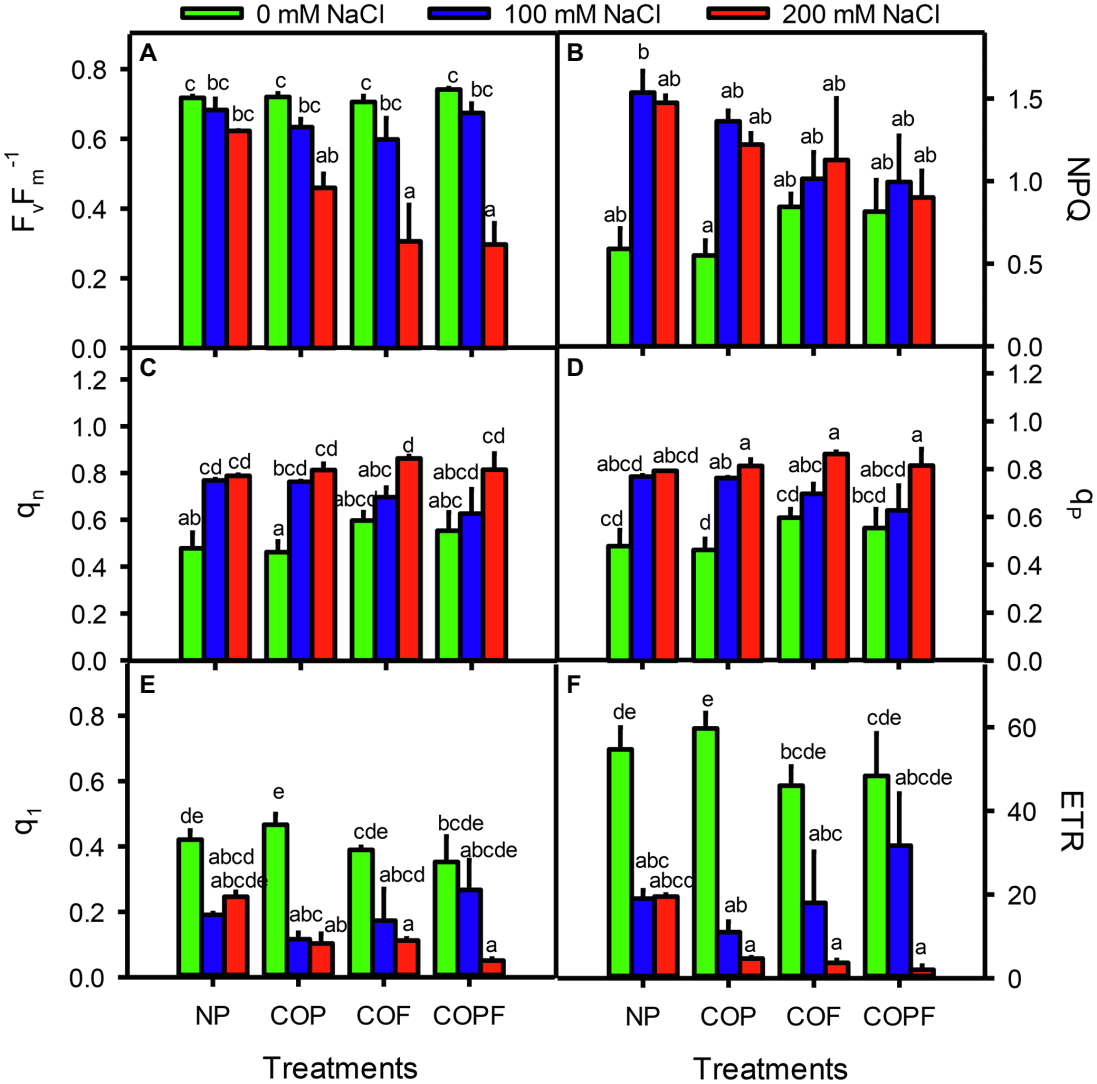
Effect of salinity and coumarin applications on Chlorophyll Fluorescence in *Sorghum bicolor* plants. F_v_/F_m_ **(A)**, NPQ **(B)**, q_n_ **(C)**, q_p_ **(D)**, q_l_ **(E)**, ETR **(F)**. Plants were treated with 0 mg L^−1^ coumarin (NP), 100 mg L^−1^ coumarin as seed priming (COP), 100 mg L^−1^ coumarin as foliar application (COF) and combined treatment (COPF), under 0, 100 and 200 mM NaCl. Values are represented as means ±SE (n = 3). Different letters represent significant differences (*p* < 0.05) by Tukey’s HSD test.

The Fv/Fm ratio was significantly decreased with increasing salinity, and COU treatments were unable to halt the declining Fv/Fm. Maximum decrease in Fv/Fm was observed in 200 mM NaCl at COF and COPF treatments. On the other hand, NPQ, qN, and qP values were increased with increasing salinity in both COU treated and untreated plants. The ql and ETR values were significantly decreased with increasing salinity in both COU treated and untreated plants (Figures 3A–F).

The Y(II) significantly decreased with increasing salinity and COU application did not help plants in improving Y(II), except COPF at 100 mM NaCl. The Y(NPQ) increased with increasing salinity, in both COU treated and untreated plants. The Y(NO) values were unchanged throughout the experiment (Table 1).

**Table 1:** Effect of salinity and coumarin applications on chlorophyll fluorescence in *Sorghum bicolor* plants.

Treatments	Y(II)	Y(NPQ)	Y(NO)
NP	0.40 ± 0.04 de	0.22 ± 0.04 ab	0.38 ± 0.01 a
NP 100	0.14 ± 0.01 abc	0.52 ± 0.00 c	0.33 ± 0.02 a
NP 200	0.14 ± 0.01 abcd	0.51 ± 0.01 bc	0.33 ± 0.01 a
COP	0.43 ± 0.02 e	0.20 ± 0.03 a	0.36 ± 0.01 a
COP 100	0.08 ± 0.02 ab	0.53 ± 0.01 c	0.39 ± 0.02 a
COP 200	0.035 ± 0.04 a	0.53 ± 0.01 c	0.43 ± 0.01a
COF	0.34 ± 0.03 bcde	0.30 ± 0.03 abc	0.36 ± 0.01a
COF 100	0.13 ± 0.09 abc	0.43 ± 0.05 abc	0.43 ± 0.05 a
COF 200	0.02 ± 0.01 a	0.48 ± 0.10 abc	0.50 ± 0.11 a
COPF	0.36 ± 0.07 cde	0.29 ± 0.07 abc	0.35 ± 0.01 a
COPF 100	0.23 ± 0.09 abcde	0.38 ± 0.10 abc	0.39 ± 0.03 a
COPF 200	0.01± 0.01 a	0.46 ± 0.05 abc	0.53 ± 0.04 a

NP (Non-prime control), NP 100 (Non-prime + salinity 100 mM), COP (Prime with Coumarin), COP 100 (Prime with Coumarin + salinity 100 mM), COP 200 (Prime with Coumarin + salinity 200 mM), COF (Coumarin foliar), COF 100 (Coumarin foliar + salinity 100 mM), COF 200 (Coumarin foliar + salinity 200 mM), COFP (Coumarin foliar and prime), COFP 100 (Coumarin foliar and prime + salinity 100 mM), COFP 200 (Coumarin foliar and prime + salinity 200 mM). Values are represented as means ± SE (n = 3). Different letters represent significant differences (p < 0.05) by Tukey’s HSD test.

### Osmolytes and Stress Markers

**Figure 4:**
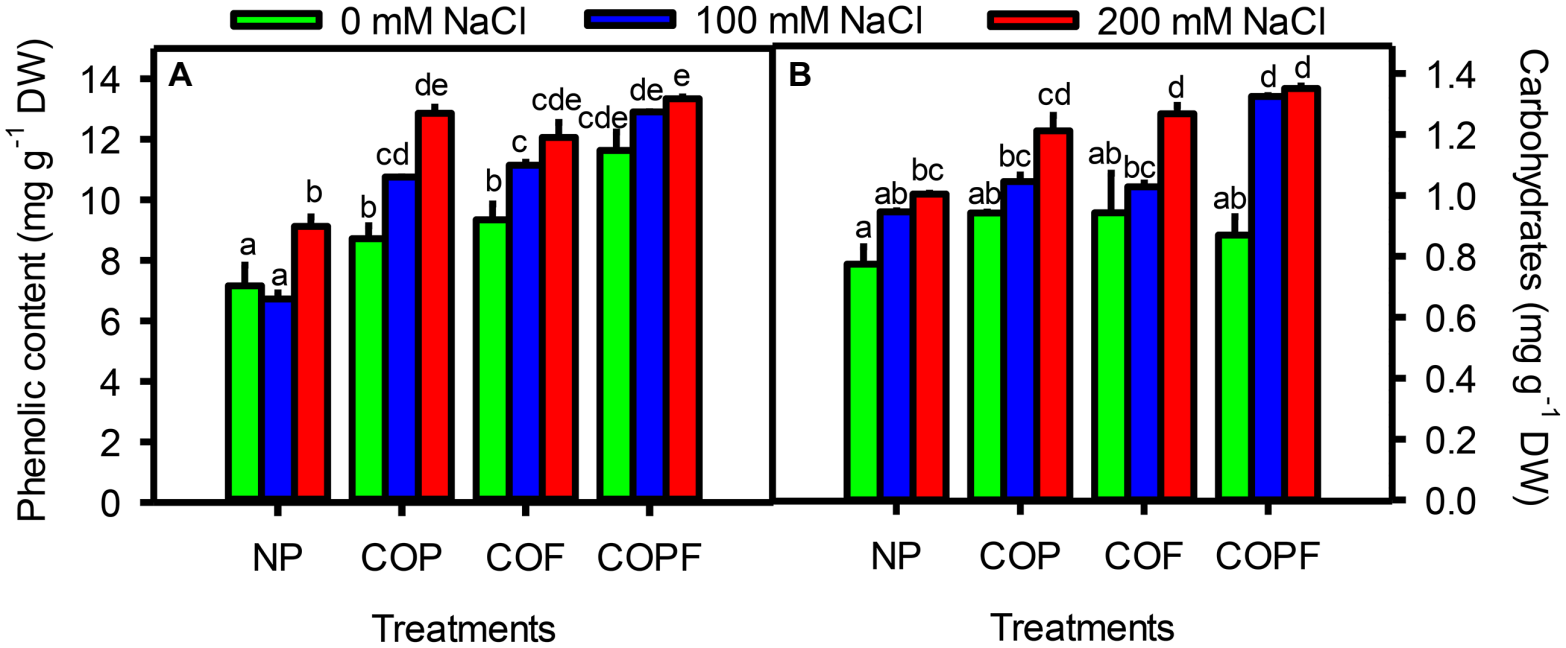
Effect of salinity and coumarin applications on Phenolic contents **(A)**, Carbohydrate contents **(B)**. Plants were treated with 0 mg L^−1^ coumarin (NP), 100 mg L^−1^ coumarin as seed priming (COP), 100 mg L^−1^ coumarin as foliar application (COF) and combined treatment (COPF), under 0, 100 and 200 mM NaCl. Values are represented as means ±SE (n = 3). Different letters represent significant differences (*p* < 0.05) by Tukey’s HSD test.

Salinity had an increasing effect on total soluble carbohydrates and phenols of Sorghum, that was further enhanced by COU applications. The maximum concentrations of carbohydrates and phenolic contents were observed in the COPF treatment, particularly at 200 mM NaCl (Figures 4A,B).

**Figure 5:**
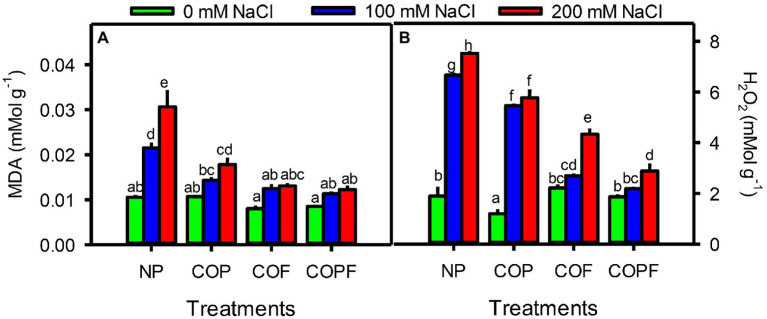
Effect of salinity and coumarin applications on stress markers MDA **(A)** and H_2_O_2_ **(B)**. Plants were treated with 0 mg L^−1^ coumarin (NP), 100 mg L^−1^ coumarin as seed priming (COP), 100 mg L^−1^ coumarin as foliar application (COF) and combined treatment (COPF), under 0, 100 and 200 mM NaCl. Values are represented as means ±SE (*n* = 3). Different letters represent significant differences (*p* < 0.05) by Tukey’s HSD test.

Damage markers in terms of malondialdehyde (MDA) and H_2_O_2_ contents were significantly (*p* < 0.01) increased with increasing salinity. The highest amount of both markers was observed at highest salinity of non-prime control plants. All COU treatments significantly (*p* < 0.01) reduced the MDA and H_2_O_2_ contents under both saline and non-saline conditions. The maximum decline was observed in COPF treatment as compared to non-prime control (Figures 5A,B).

### Antioxidant Enzyme Activities

An inverse relation of protein contents was observed with increasing salinity treatments. Maximum decrease in protein content was found at highest salinity (200 mM NaCl). However, coumarin-treated plants showed significant (*p* < 0.001) increase in protein contents under saline conditions as compared to control plants. The maximum increase in protein content was recorded in both COP and COF treatments.

**Figure 6:**
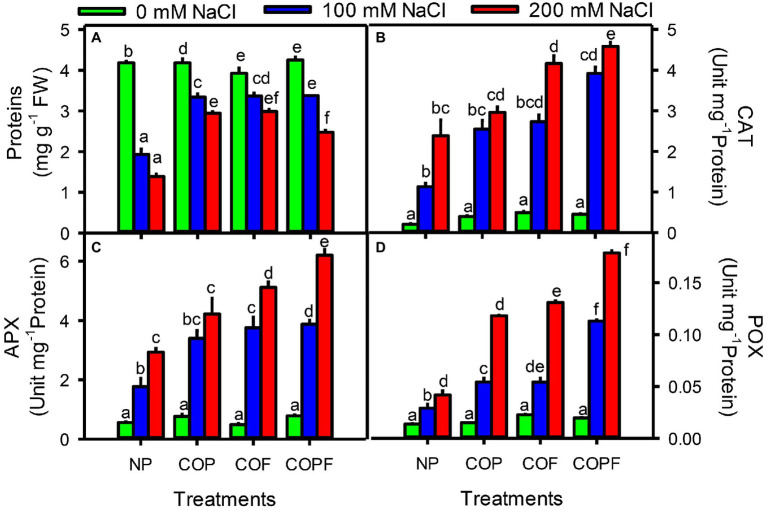
Effect of salinity and coumarin applications on protein contents and antioxidant enzymes activities. Protein **(A)**, Catalase **(B)**, Ascorbate Peroxidase **(C)** and Guaicol peroxidase **(D)**. Plants were treated with 0 mg L^−1^ coumarin (NP), 100 mg L^−1^ coumarin as seed priming (COP), 100 mg L^−1^ coumarin as foliar application (COF) and combined treatment (COPF), under 0, 100 and 200 mM NaCl. Values are represented as means ±SE (*n* = 3). Different letters represent significant differences (*p* < 0.05) by Tukey’s HSD test.

Antioxidant enzymes activities including CAT, APX, and GPX were significantly increased (*p* < 0.001) with increasing salinity treatments throughout the experiment. However, all COU treatments (COP, COF, and COPF) further aided the antioxidant enzyme activities. The maximum enzymatic activities of CAT, APX, and GPX were observed at highest salinity in the COPF treatment as compared to control and other COU treatments (Figures 6A–D).

## Discussion

A two-way ANOVA indicated that the salinity stress and COU treatments had significant effects on plant growth, biochemical parameters, photosynthetic efficiency, and antioxidant enzymes activities of Sorghum. The growth reduction (shoot and root lengths, fresh, and dry weights) could be due to increase in osmotic, ionic, and oxidative stresses, which are reported to inhibit various physiological, morphological, and anatomical processes of plants (Siddiqui et al., 2022). On the other hand, COU application sharply stimulated the overall growth performance under salinity and the combined treatment (COPF) found to be most effective in alleviating salinity-induced toxic effects. Previously, it was reported that COU pretreatment improved shoot height, biomass production, moisture content, and net assimilation rate in many crops including *Vicia faba* (Saleh et al., 2015), rice (Rahman et al., 2016), cucumber (Mohsin et al., 2019), and in tomato (Parvin et al., 2020). These effects are mainly due to effective osmoregulation (by osmolytes and osmoprotectants), ionic balance, and antioxidant defense system. It is also reported that endogenous COU level is positively correlated with plants growth regulators (Gurmani et al., 2011). COU reported to interfere with the biosynthesis of auxin to promote shoot and root growth and branching to attain efficient light and water (Lupini et al., 2014). In present study, improvement in shoot and root growth by COPF indicated toward the enhanced endogenous level of COU (Cheynier et al., 2013). COU has a strong antioxidant nature, which may regulate plant growth by showing effective antioxidant capacity (Parvin et al., 2021). COU also possess gibberellin-like activity and can stimulate amylase biosynthesis in germinating seeds (of wheat) and increase stem length in pea seedlings by inhibiting paclobutrazol (a GA inhibitor) activity (Saleh et al., 2015; Saleh and El-Soud, 2015).

Salinity stress also reduces photosynthetic efficiency by triggering hyperosmotic and oxidative damages to the photosynthetic apparatus, interrupting stomatal regulation, and reducing CO_2_ fixation (Siddiqui et al., 2020). The salt-induced reduction in chlorophylls might be due to enhanced chlorophyllase activity (Rahman et al., 2016). Present study showed that the COU application intensified the leaf pigments in Sorghum, of which COPF-treated plants had more pronounced effects under both non-saline and saline conditions. Increase in chlorophylls and other pigments ultimately enhance the food manufacturing process and in turn plant growth and biomass (Yan et al., 2016). Higher levels of leaf pigments by exogenous application of coumarin were also reported in tomato (Parvin et al., 2021) and in wheat (Saleh and Madany, 2015). Carotenoids and anthocyanins are involved in mitigating the deleterious effects of ROS in plants; therefore, higher contents of carotenoids and anthocyanins in COPF treatment indicating better heat dissipating and free radical quenching ability at chloroplast level (Winkel-Shirley, 2002). Conversely, plants with reduced carotenoid content did not have such protections, hence fragile photosynthetic activity with oxidative burst lead to growth reduction under salinity.

The exaggerated operational system of photosystem II has been reported under salinity stress (Tavakkoli et al., 2010). The analysis of chlorophyll fluorescence of the COU-treated and non-treated plants revealed that enhanced NPQ, especially at 100 mM NaCl, helped plants to dissipate excessive energy that cannot be used in photochemical reactions (Guidi et al., 2007) and may help Sorghum prevent photoinhibition of PSII (Jajoo et al., 2014). In contrast, lower NPQ values in COU-treated plants demonstrate less requirement of heat dissipation as most of the energy was channeled to photochemical quenching (qP), ETR, and Y(II) under all salinities. This was also evident by lower oxidative damage (MDA and H_2_O_2_ content) in Sorghum leaves. Similarly, a greater increase in Fv/Fm in COU-treated plants suggest more energy assimilation and conversion into ATP and NADPH, which are ultimately used for biomass production and defense systems of Sorghum under saline conditions (Hussain et al., 2020b). Excessive accumulation of toxic ions such as Na^+^ and Cl^−^ within the cytosol generally reduces photosynthetic efficiency by damaging the reaction center of PSII (Netondo et al., 2004). In our study, this phenomenon is evident in non-COU-treated plants, in which salinity-induced inhibition of ETR indicates possible disruption of PSII (Allahverdiyev et al., 2011). Therefore, it is suggested that COPF application is found beneficial for ameliorating the hazardous effects of salinity on PSII system, resulting in improved photosynthetic efficacy, enhanced ETR activity, and photochemical quenching of Sorghum plants.

Accumulation of phenolic compounds helps plants to overcome adverse effects of abiotic stresses, e.g., salinity (Pereira, 2016). Our results showed a significant increase in total phenolic content of COU-treated plants, especially in COPF treatment. The logical consequences regarding higher accumulation of phenols indicate the possible involvement of COU in phenyl-propanoid pathway. COU can activate PAL activity, thereby boosting the synthesis of phenolic compounds that scavenge harmful free radicals, as reported in Sunflower leaves (Al-Wakeel et al., 2013). In addition, COU itself is an antioxidant that directly acts to neutralize ROS and also produce other substrates that can be utilized for various physiological processes (Michalak, 2006). The salinity-induced modification in phenolic content is also reported by Bourgou et al. (2010) in black cumin which indicates its beneficial role to alleviate the negative effects of salinity. A synergistic interaction of phenolic compounds along with soluble sugars involved in integrated redox system to alleviate the oxidative damages produced by ROS by providing effective quenching system particularly in the organelles or tissues with higher soluble carbohydrates (Bolouri-Moghaddam et al., 2010).

Present investigation revealed that with the intensification of salinity the accumulation of total soluble carbohydrates increased in both COU-treated and untreated plants. However maximum increase was observed in COU application under saline conditions. Similar results were also reported in wheat seedling in which COU application significantly increases the carbohydrates accumulation, may be due to the increased amylolytic activity under stressed conditions (Saleh and Madany, 2015). Additionally, GA_3_ like activity of COU was also reported by which its exogenous application is positively correlated with sugar-enhancing ability in leaf tissues (Iqbal et al., 2012). An increase in photosynthetic activity was also observed in COU-treated plants, which may be a reason for the higher accumulation of soluble carbohydrates under saline conditions (Parvin et al., 2021). The carbohydrates accumulation in cytosol has a vital role in osmotic adjustments, carbon storage, metabolite precursors, and source of energy and played a pleiotropic role in plant growth, development, and metabolism (Rolland et al., 2002). Soluble carbohydrates such as sucrose, raffinose, and trehalose are also considered as compatible solutes and actively involved in osmoregulation during stressful condition (Ende and Peshev, 2013). Small sugars like glucose, fructose, and sucrose also involved as secondary messengers in signal transduction pathways during stress resistance (Bolouri-Moghaddam et al., 2010). Additionally fructans may act as cytosolic antioxidant processes to strengthen the antioxidant defense during stressed conditions (Afzal et al., 2021). The higher accumulation of carbohydrates by COU pretreatment was also reported in sunflower and faba bean (Al-Wakeel et al., 2013; Saleh et al., 2015). Some other phenolic substance such as ferulic acid was also reported to enhance carbohydrates in leaves under stressful conditions (Li et al., 2013).

Increased contents of stress markers like MDA and H_2_O_2_ are indicative of salinity-induced oxidative stress in Sorghum (Siddiqui et al., 2022). However, COU application significantly decreases MDA and H_2_O_2_ contents under saline conditions. Phenolic compounds (e.g., coumarin) have generally strong antioxidant nature that can protect ROS-medicated injuries of cellular components and membranes, especially under stress (Yan et al., 2017). It can be assumed that COU application seems to help in maintaining the integrity of plasma membrane by reducing MDA content which indicates reduction in membrane injuries of sorghum under salinity. The role of phenolic compounds in membrane integrity is also reported by Arora et al. (2000). COU have the ability to bind and accumulate the polar ends of phospholipids that stabilize and strengthen the integrity of the plasma membrane and cell wall (Arora et al., 2000). It also helps plant to increase endogenous cell wall-bound phenolic contents that enhance the activities of peroxidases to maintain extensibility and strength of cell wall under stress (Haghighi et al., 2014).

The variation in soluble protein content with increasing salinity has a considerable role in maintaining the plants cellular activities and nitrogen storage. In this study, decrease in protein content indicates toward the reduction in protein synthesis and/or increased proteolysis under salinity stress (Dubey, 1999; Alamri et al., 2021). However, COU treatment significantly slowdowns the declining levels of total proteins suggesting a role of COU priming-mediated protease activity in Sorghum seeds, which has been reported in *Arabidopsis thaliana* (Araniti et al., 2017). To alleviate the oxidative damages caused by ROS, activation of antioxidant defense system provides an efficient strategy to withstand under stressful conditions. Both enzymatic and non-enzymatic antioxidant system played an important role to induce salinity tolerance in plants. The enzymatic antioxidant system of sorghum was activated under both COU-treated and untreated plants to cope with salinity-induced oxidative stress. However, activities of CAT, APX, and GPX were enhanced many folds in COU-treated plants. Therefore, it can be speculated that COU play an important role to strengthen the antioxidant defense system against oxidative damages (Saleh et al., 2015). Various phenolic substances such as Coumarin, ferulic, ellagic, and cinnamic acids have been reported to involved in antioxidant defense system by increasing antioxidant enzymes (CAT, APX, and GPX) under normal and stressed conditions (Li et al., 2013; Singh et al., 2017).

The higher levels of antioxidant enzymes by COU pretreatment in seedlings of *Sorghum bicolor* were also reported in our previous study (Sultana et al., 2020). Similar results were also reported in wheat seedlings by COU application (Saleh and Madany, 2015). The involvement of phenolic compounds (Vanillic acid and Quercetin) in antioxidant defense mechanism was also reported in tomato plant under saline conditions (Parvin et al., 2020). The involvement of phenolic compound in detoxification of ROS by activating antioxidant defense system was also reported by Singh et al. (2019). Such enhancements could be considered a better strategy to resist salinity-induced negative effects on metabolic processes and plant growth.

## Conclusion

Our results indicated that exogenous application of COU significantly enhanced salinity resistance and increased the growth of *Sorghum bicolor* under saline conditions. It modulates concentrations of osmolytes, leaf pigments, and photosynthetic efficiency. COU applications prevent PSII reaction centers by inducing carotenoids and anthocyanins and dissipating excessive energy. Salinity-induced oxidative damages to cellular components and membranes (as indicated by damage makers) were also minimized by activating enzymatic and non-enzymatic antioxidant defense system. The combined application of primed and foliar spray (COPF) displayed better results than their individual ones. Therefore, COPF represents an efficient mode of COU application, which can be utilized for growth improvement and biomass production of a multipurpose crop *Sorghum bicolor* from saline degraded lands. However, the effect of COU on endogenous hormones and accurate estimation of their level of induction as well as the molecular modifications underlying COU biosynthesis needs to be evaluated in future studies.

## Data Availability Statement

The original contributions presented in the study are included in the article/supplementary material, further inquiries can be directed to the corresponding authors.

## Author Contributions

MA and MQ conceived the idea and helped in manuscript writing and proofreading. RS executed the idea and conducted the experiments. XW and AM provided technical expertise. RS, SF, and TH helped in data collection and analyses. All authors have read the manuscript, helped in revision, and approved the article.

## Funding

The publication of the present work is supported by the Natural Science Basic Research Program of Shaanxi Province (grant no. 2018JQ5218), the National Natural Science Foundation of China (51809224), and Top Young Talents of Shaanxi Special Support Program.

## Conflict of Interest

The authors declare that the research was conducted in the absence of any commercial or financial relationships that could be construed as a potential conflict of interest.

## Publisher’s Note

All claims expressed in this article are solely those of the authors and do not necessarily represent those of their affiliated organizations, or those of the publisher, the editors and the reviewers. Any product that may be evaluated in this article, or claim that may be made by its manufacturer, is not guaranteed or endorsed by the publisher.
